# Risk Factors for 30-Day Unplanned Re-Operation in Pediatric Upper Extremity Surgery: A National Surgical Quality Improvement Program (NSQIP)-Pediatric Analysis

**DOI:** 10.7759/cureus.38140

**Published:** 2023-04-26

**Authors:** Anthony K Chiu, Theodore Quan, Denver Kraft, Sean Tabaie

**Affiliations:** 1 Orthopaedic Surgery, George Washington University School of Medicine and Health Sciences, Washington, D.C., USA; 2 Orthopaedic Surgery, Georgetown University School of Medicine, Washington, D.C., USA; 3 Orthopaedic Surgery, Children's National Hospital, Washington, D.C., USA

**Keywords:** nsqip peds, unplanned reoperation, risk factors, pediatrics, orthopedic surgery, upper extremity

## Abstract

Introduction

The unplanned re-operation rate has been used as one marker of procedure quality in numerous surgical sub-fields. The purpose of this study was to determine independent risk factors for unplanned re-operations within 30 days following pediatric upper extremity surgery.

Methods

Pediatric patients who had a primary upper extremity procedure from 2012 to 2019 were identified in the National Surgical Quality Improvement Program-Pediatric database. The procedures included percutaneous fixation of supracondylar humerus fractures, open treatment of humeral condylar fractures, tendon sheath incision, repair of syndactyly, and reconstruction of polydactyly. Patients were categorized by those who had unplanned return to the operating room within 30 days and patients who did not. Patient demographics, clinical characteristics, and medical co-morbidities were evaluated for their association with re-operation using bivariate and multivariate analysis.

Results

A total of 27,536 pediatric patients underwent primary upper extremity surgeries; of these, 290 (1.1%) required an unplanned re-operation. After controlling for potential confounding variables on multivariable regression analysis, American Society of Anesthesiologists (ASA) class III-V (OR 15.89; p<0.001), inpatient procedure (OR 1.29; p=0.044), emergent/urgent triage (OR 3.75; p<0.001), longer operative time (OR 1.01; p<0.001), and prolonged hospital stay (OR 1.01; p=0.010) were independent predictors for re-operation.

Conclusion

This study demonstrates that the national rate of 30-day unplanned re-operation in pediatric upper extremity surgeries is low overall. The greatest risk factors for unplanned re-operation were ASA class III-V, inpatient setting, emergent/urgent triage, longer operative time, and prolonged hospital stay. This knowledge can help further improve patient outcomes through risk stratification and preoperative planning.

## Introduction

The unplanned re-operation rate has garnered increased attention as a metric for surgical quality in orthopedic surgery as well as numerous other surgical fields [1]. Traditionally, mortality rates and the rates of specific complications have been looked at to assess quality. However, using complication rates to assess quality may be confounded by the inherent variability in surgical procedures as well as heterogeneous patient populations between hospitals [2], and the mortality rates for most surgical procedures are very low [3].

Unplanned re-operations are associated with poorer outcomes, increased burden on the healthcare system, and increased morbidity and mortality [1,4]. The unplanned re-operation rate is generalizable, objective, quantifiable, and reliably documented [5]. Its generalizability makes it especially fitting for evaluating quality in groups of procedures. Unplanned re-operations are associated with preventable causes of harm to the patient [6], resulting in the addition of the 30-day unplanned re-operation rate to the National Surgical Quality Improvement Program (NSQIP) database by the American College of Surgeons (ACS) and its adoption as a broadly applicable index for surgical quality [6]. Identification of risk factors for unplanned re-operations may allow for improved surgical quality through preoperative risk stratification and increased surgeon awareness.

The unplanned re-operation rate has been studied in many surgical sub-fields, including general surgery [7], vascular surgery [8], plastic surgery [9], neurosurgery [10-12], and orthopedic surgery [13-15]. There are studies evaluating unplanned re-operation rates in adult upper extremity surgery [16], but few focused on the pediatric population. Thibaudeau et al. investigated 30-day readmission and re-operation rates in the most common pediatric upper extremity surgeries using data from 2012-2014 [17]. Surgical site infection, failure to thrive, central nervous system (CNS) history, cardiac history, gastrointestinal history, respiratory history, and inpatient procedures were all found to be associated with 30-day readmission, but associations with unplanned re-operation were not reported.

A large database study is fitting for an investigation into unplanned re-operation rates given the low incidence of re-operation in the pediatric population. The purpose of this study was to identify risk factors for unplanned re-operation using the ACS NSQIP-Pediatric (ACS NSQIP-P), facilitating improved surgical quality through preoperative risk stratification and planning. We hypothesized that pediatric upper extremity surgeries would be overall safe and effective with discrete identifiable risk factors for re-operation.

## Materials and methods

The ACS NSQIP-P database was used to conduct this retrospective analysis from 2012-2019. The NSQIP-P registry has been used frequently to study outcomes following various surgical procedures [18,19]. All patient information is de-identified [20].

Patient selection

Current procedural terminology (CPT) codes were used to identify primary upper extremity procedures. These CPT codes included 24538 (percutaneous skeletal fixation of supracondylar or transcondylar humerus fractures), 24579 (open treatment of humeral condylar fracture), 26055 (tendon sheath incision), 26560, 26561, 26562 (repair of simple and complex syndactyly), and 26587 (reconstruction of polydactyly). These codes are consistent with prior studies [17]. Only patients less than 18 years were included in this study. Both inpatient and outpatient procedures were included. Two patient groups were categorized: patients who required an unplanned return to the operating room (re-operation) within 30 days of their primary procedure and patients who did not require re-operation.

Baseline characteristics

Various patient characteristics were assessed to evaluate the association of risk factors with unplanned re-operation. Demographics and clinical characteristics included gender, race, American Society of Anesthesiologists (ASA) classification, procedure type (inpatient or outpatient), anesthesia type (general or non-general), triage (elective or emergent/urgent), age, preoperative albumin, preoperative sodium, and preoperative hematocrit. Operative time and length of hospital stay were also assessed. Co-morbidities were grouped into clinically relevant categories. Pulmonary co-morbidities included a history of asthma, oxygen support, structural pulmonary/airway abnormalities, tracheostomy, bronchopulmonary dysplasia, chronic lung disease, and ventilator dependence. Cardiac co-morbidities included any cardiac risk factors, previous cardiac surgery, cardiopulmonary resuscitation within seven days of surgery, and inotropic support at the time of surgery. Neurological co-morbidities included seizure disorder, developmental delay, impaired cognitive status, structural CNS abnormality, cerebral palsy, and neuromuscular disorder. Gastrointestinal co-morbidities included esophageal, gastric, and intestinal disease. Biliary co-morbidities included biliary, liver, and pancreatic disease. Immune disease, steroid use within 30 days of surgery, failure to thrive, nutritional support, bleeding or hematologic disorder, and bone marrow transplant were further recorded.

Statistical analysis

Demographic and co-morbidity variables were analyzed using Pearson’s Chi-squared test and analysis of variance. Variables with a p-value < 0.05 were selected for multivariable regression analysis. Multivariable logistic regression analysis was performed to identify the risk factors that were independently associated with re-operation. Odds ratios with 95% confidence intervals were reported for the regression analysis results. A sub-analysis was conducted, which reported unplanned re-operation rates and multiple logistic regression analyses stratified by procedure type. All analyses were performed using IBM SPSS Statistics for Windows, Version 28.0 (Released 2021; IBM Corp., Armonk, New York, United States). The threshold for statistical significance was < 0.05 for all analyses.

## Results

A total of 27,536 pediatric patients underwent primary upper extremity surgeries and were included in the analysis. Of these, 27,246 patients (98.9%) did not have an unplanned return to the operating room whereas 290 (1.1%) required an unplanned re-operation.

When comparing the patient demographics and clinical characteristics between the two cohorts, on bivariate analysis, an ASA class of III, IV, or V (p<0.001), an inpatient procedure (p<0.001), and an emergent/urgent procedure (p<0.001) were significantly associated with unplanned re-operation. A longer operative time (p<0.001) and a prolonged length of hospital stay (p<0.001) were also associated with re-operation (Table 1). Pulmonary co-morbidities (p=0.013) and gastrointestinal co-morbidities (p=0.005) were also associated with re-operation on bivariate analysis (Table 2).

**Table 1:** Demographics and Clinical Characteristics for Upper Extremity Surgical Patients (Pediatric) *Pearson’s chi-squared test; **Analysis of variance Bolding equals significance p<0.05 ASA, American Society of Anesthesiologists; SD, standard deviation

Variables	No Re-operation	Re-operation	P-value
Total patients, n	27,246	290	
Sex, n (%)			0.546^*^
Female	12,604 (46.3)	129 (44.5)	
Male	14,642 (53.7)	161 (55.5)	
Race, n (%)			0.278^*^
White	15,104 (55.4)	170 (58.6)	
Non-White	12,142 (44.6)	120 (41.4)	
ASA, n (%)			< 0.001^*^
I or II	26,648 (97.8)	224 (77.2)	
III, IV or V	598 (2.2)	66 (22.8)	
Procedure, n (%)			< 0.001^*^
Outpatient	18,616 (68.3)	153 (52.8)	
Inpatient	8,630 (31.7)	137 (47.2)	
Anesthesia Type, n (%)			0.465^*^
General	27,195 (99.8)	290 (100.0)	
Non-General	50 (0.2)	0 (0.0)	
Triage, n (%)			< 0.001^*^
Elective	11,719 (43.0)	61 (21.0)	
Emergent/Urgent	15,527 (57.0)	229 (79.0)	
Mean age, years (SD)	5.29 (2.94)	5.51 (2.76)	0.213^**^
Preoperative albumin, g/dL (SD)	4.16 (0.45)	4.20 (0.33)	0.671^**^
Preoperative sodium, mEq/L (SD)	138.40 (2.38)	138.02 (2.31)	0.254^**^
Preoperative hematocrit, % (SD)	34.91 (3.06)	34.71 (2.78)	0.636^**^
Operative time, minutes (SD)	41.87 (40.61)	52.51 (45.83)	< 0.001^**^
Length of stay, days (SD)	0.68 (3.71)	1.63 (3.81)	< 0.001^**^

**Table 2:** Co-morbidities Among Upper Extremity Surgical Patients *Pearson’s chi-squared test Bolding equals significance p<0.05

Co-morbidities	No Re-operation	Re-operation	P-value^*^
Total patients, n	27,246	290	
Pulmonary co-morbidity, n (%)	1,231 (4.5)	22 (7.6)	0.013
Cardiac co-morbidity, n (%)	599 (2.2)	5 (1.7)	0.583
Neurological co-morbidity, n (%)	1,035 (3.8)	17 (5.9)	0.068
Gastrointestinal co-morbidity, n (%)	399 (1.5)	10 (3.4)	0.005
Biliary co-morbidity, n (%)	7 (0.0)	0 (0.0)	0.824
Immune disease, n (%)	8 (0.1)	0 (0.0)	0.813
Steroid use, n (%)	81 (0.3)	0 (0.0)	0.352
Failure to thrive, n (%)	29 (0.2)	0 (0.0)	0.659
Nutritional support, n (%)	93 (0.3)	0 (0.0)	0.319
Bleeding disorder, n (%)	8 (0.1)	0 (0.0)	0.813
Hematologic disorder, n (%)	116 (0.4)	1 (0.3)	0.833
Bone marrow transplant, n (%)	5 (0.0)	0 (0.0)	0.851

After controlling for potential confounding variables on multivariable logistic regression analysis, ASA class of III-V (OR 15.892, 95%CI 11.600-21.771; p<0.001), inpatient procedure (OR 1.289, 95%CI 1.007-1.649; p=0.044), emergent/urgent procedure (OR 3.747, 95%CI 2.714-5.172; p<0.001), longer operative time (OR 1.004, 95%CI 1.002-1.006; p<0.001), and prolonged hospital stay (OR 1.012, 95%CI 1.003-1.021; p=0.010) were found to be independent risk factors for unplanned re-operation (Table 3).

**Table 3:** Multivariable Regression Analysis of Risk Factors Associated with Re-operation ^a ^ASA III, IV, or V compared to ASA I or II; ^b ^Inpatient compared to outpatient; ^c ^Emergent/urgent compared to elective Bolding equals significance p<0.05 CI, confidence interval; ASA, American Society of Anesthesiologists

Variables	Odds Ratio	95% CI		P-Value
		lower	higher	
ASA^a^	15.892	11.600	21.771	< 0.001
Procedure^b^	1.289	1.007	1.649	0.044
Triage^c^	3.747	2.714	5.172	< 0.001
Operative time	1.004	1.002	1.006	< 0.001
Length of stay	1.012	1.003	1.021	0.010
Pulmonary co-morbidity	0.929	0.568	1.518	0.769
Gastrointestinal co-morbidity	0.746	0.352	1.580	0.444

Sub-analysis of the overall study population revealed the unplanned re-operation rates by procedure. Percutaneous skeletal fixation of supracondylar or transcondylar humerus fractures had an unplanned re-operation rate of 1.2%. Open treatment of humeral condylar fractures had an unplanned re-operation rate of 1.6%. Tendon sheath incision had an unplanned re-operation rate of 0.2%. Repair of simple or complex syndactyly had an unplanned re-operation rate of 0.5%. Reconstruction of polydactyly had an unplanned re-operation rate of 0.3%. For percutaneous skeletal fixation of supracondylar or transcondylar humerus fractures, ASA class of III-V (OR 22.258, 95%CI 15.799-31.357; p<0.001) and emergent/urgent procedure (OR 1.902, 95%CI 1.280-2.826; p=0.001) were found to be independent risk factors for re-operation. For open treatment of humeral condylar fracture, ASA class of III-V (OR 16.337, 95%CI 6.396-41.732; p<0.001), emergent/urgent procedure (OR 2.117, 95%CI 1.014-4.423; p=0.046), and hospital length of stay (OR 1.056, 95%CI 1.005-1.109; p = 0.031) were found to be independent risk factors for re-operation. The remaining procedures did not reveal any significant risk factors (Table 4).

**Table 4:** Sub-Analysis of Re-operations, Unplanned Re-operation Rates, and Multivariable Logistic Regression Analysis ^a^ASA III, IV, or V compared to ASA I or II; ^b^Emergent/Urgent compared to Elective; *No risk factors found to be significant Bolding equals significance p<0.05 CI, confidence interval; ASA, American Society of Anesthesiologists; CPT, current procedural terminology

Sub-Group	Unplanned Re-operation Rate (%)	Odds Ratio	95% CI		P-Value
			lower	higher	
CPT-24538: Percutaneous skeletal fixation of supracondylar or transcondylar humerus fractures	1.2				
ASA^a^		22.258	15.799	31.357	<0.001
Triage^b^		1.902	1.280	2.826	0.001
CPT-24579: Open treatment of humeral condylar fracture	1.6				
ASA^a^		16.337	6.396	41.732	<0.001
Triage^b^		2.117	1.014	4.423	0.046
Length of stay		1.056	1.005	1.109	0.031
CPT-26055: Tendon sheath incision^*^	0.2				
CPT-26560, 26561, 26562: Repair of simple and complex syndactyly^*^	0.5				
CPT-26587: Reconstruction of polydactyly^*^	0.3				

## Discussion

This study was designed to identify risk factors for unplanned re-operation within 30 days for the most common pediatric upper extremity. The unplanned re-operation rate has gained traction as a valuable metric for determining surgical quality. In this retrospective analysis, the rate of unplanned re-operation in pediatric upper extremity procedures was low, 1.1%, consistent with a prior report, which documented a 0.7% unplanned re-operation rate [17]. Independent risk factors for unplanned re-operation were an ASA classification of III-V, emergent/urgent triage (vs. elective procedures), inpatient setting, longer operative time, and prolonged hospital stay.

ASA classification III-V was the greatest independent predictor of unplanned re-operation. Higher ASA classification is known to be associated with increased re-operation rates in prior studies in the orthopedic literature [21,22]. The ASA classification has been used since the 1940s to provide a categorization of operative risk; however, there are limitations including low inter-reporter reliability, vague criteria, and simplicity [23]. Despite these limitations, the ASA classification is a well-known score that can easily provide orthopedic surgeons with an objective indicator of unplanned re-operation risk.

Other independent predictors of unplanned re-operation were emergent/urgent triage and inpatient setting. Emergent/urgent triage is known to carry higher rates of morbidity, mortality, and unplanned re-operation in surgical procedures [24,25]. It has been argued in the prior literature that procedures with this triage status should be considered separately when measuring quality, as there are inherently higher rates of complications [26]. Therefore, the higher unplanned re-operation rate in emergent/urgent pediatric upper extremity procedures found in this study may be interpreted as inherent to the procedure type, rather than being due to differences in quality. Further research may investigate the causes of unplanned re-operation in emergent/urgent procedures. Regarding the inpatient vs. outpatient setting, there has been a recent trend toward outpatient surgery in many orthopedic subspecialties [27-29]. In the pediatric population, Makarewich et al. demonstrated that the shift to outpatient surgery in type II supracondylar humerus fractures has the potential to reduce cost, with no change in outcomes [30]. Modest et al. subsequently generated similar findings after retrospectively analyzing over 8,000 pediatric patients who underwent operative management of supracondylar humerus fractures [29]. However, it is difficult to discern whether the association between unplanned re-operation and outpatient procedures is due to differences in case selection, patient co-morbidities, or other factors.

Finally, longer operative times and prolonged hospital stays were associated with unplanned re-operation. A similar association has been reported in other surgical procedures [5]. It is unclear whether this finding is due to independent causation, confounding effects, or effect modification from another variable. Although the data in this study cannot show causation, it is sensible that patients who had an intraoperative complication would subsequently require longer operative times and hospital stays.

Limitations

There are several limitations to this study that must be taken into account. First, this research is conducted through the querying of CPT codes. Although CPT codes are frequently used in the medical literature, they were created primarily for billing purposes rather than for research. Thus, medical coding groups heterogeneous procedures and often does not capture the granularity of information that clinicians are interested in. Additionally, our study analyzed the most common pediatric upper extremity procedures, and therefore cannot be applied to procedures not included in the methodology. Next, this research analyzes only the 30-day re-operation rate which is tracked by ACS NSQIP-P. Although this is the most frequently analyzed time period used for measuring quality, it has been previously demonstrated in the literature that the majority of re-operations, globally, in orthopedic surgery occur beyond the 30-day mark [1]. Therefore, this rate likely does not capture the full extent of unplanned re-operations which occur outside of this time frame. The 30-day period may be better at capturing wound complications (surgical site infection, inadequate wound closure, anastomosis), rather than the mechanical complications of surgery [1]. While this study assumes that the 30-day re-operation rate is a valuable proxy for surgical quality, this data does not report other clinically important outcomes such as patient satisfaction, chronic pain, and functionality, which are not available from ACS NSQIP-P. Future studies may look to evaluate longer post-operative time periods for unplanned re-operation. Lastly, ACS-NSQIP-P is limited to participating centers, which contain a higher proportion of academic hospitals and therefore may not be generalizable to all patient populations.

## Conclusions

The results of this study revealed that the national rate of unplanned reoperation for the most common pediatric upper extremity surgeries is 1.1%. Independent risk factors for unplanned reoperation were ASA classification of III-V, emergent/urgent triage (vs. elective procedures), inpatient setting, longer operative time, and prolonged hospital stay. The information gained from this study may facilitate further improvement in surgical quality through pre-operative risk stratification and surgeon awareness of adverse outcomes.
